# Diagnostic value of using a combination of nucleic acid and specific antibody tests for SARS-CoV-2 in coronavirus disease 2019

**DOI:** 10.1017/S0950268821000406

**Published:** 2021-02-17

**Authors:** Kaochang Zhao, Li Ai, Yang Zhao, Tao Wang, Zhishui Zheng, Shaolin Zeng, Xuhong Ding, Suping Hu, Hanxiang Nie

**Affiliations:** 1 Department of Respiratory & Critical Medicine, Renmin Hospital of Wuhan University, Wuhan 430060, China; 2 Department of Nephrology, the Third People's Hospital of Hubei Province, Wuhan 430033, China

**Keywords:** Antibody, COVID-19, nucleic acid, SARS-CoV-2

## Abstract

Coronavirus disease 2019 (COVID-19) is a newly emerged disease with various clinical manifestations and imaging features. The diagnosis of COVID-19 depends on a positive nucleic acid amplification test by real-time reverse transcription-polymerase chain reaction (RT-PCR) for severe acute respiratory syndrome coronavirus 2 (SARS-CoV-2). However, the clinical manifestations and imaging features of COVID-19 are non-specific, and nucleic acid test for SARS-CoV-2 can have false-negative results. It is presently believed that detection of specific antibodies to SARS-CoV-2 is an effective screening and diagnostic indicator for SARS-CoV-2 infection. Thus, a combination of nucleic acid and specific antibody tests for SARS-CoV-2 will be more effective to diagnose COVID-19, especially to exclude suspected cases.

## Background

Since 8 December 2019, an increasing number of patients with coronavirus disease 2019 (COVID-19) have been reported in Wuhan, China [1–3]. After the recognition of COVID-19, there has been an exponential rise in the number of cases in more than 195 countries worldwide [4]. The causative pathogen has been confirmed as a novel enveloped RNA betacoronavirus [5] that is now known as severe acute respiratory syndrome coronavirus 2 (SARS-CoV-2), which is phylogenetically related to the SARS-CoV [6]. Accurate diagnosis of SARS-CoV-2 infection is essential for preventing virus transmission and assuring timely treatment of patients. Patients with epidemiological contact history, severe acute respiratory infection and no other aetiology that fully explains the clinical presentation can be diagnosed as suspected COVID-19 [7]. The clinical manifestations and imaging features of COVID-19 are often non-specific, therefore making it difficult to distinguish between COVID-19 and other types of pneumonia just based on these features [8]. Consequently, patients with other respiratory pathogen infection may be misdiagnosed as suspected cases of COVID-19. According to World Health Organisation (WHO) interim guidance, the COVID-19 must be confirmed by detection of SARS-CoV-2 nucleic acid via real-time reverse transcription-polymerase chain reaction (RT-PCR) [7]. However, nucleic acid test for SARS-CoV-2 can have false-negative results due to various reasons. Therefore, it is challenging to confirm or exclude coronavirus infection in those suspected cases. Antibody detection of SARS-CoV-2 has been reported as an important mean to assist nucleic acid diagnosis and rapid screening [9]. In this mini-review, we aimed to describe the value of a combined examination of nucleic acid and specific antibody for SARS-CoV-2 in the diagnosis of COVID-19, particularly those suspected cases.

## Nucleic acid test for SARS-CoV-2

The viral nucleic acid test on respiratory specimens using RT-PCR assay is considered as the gold standard in the diagnosis of SARS-CoV-2 infection and is also used as an indicator for isolating, discharging and transferring patients diagnosed with COVID-19 [7, 10, 11]. Therefore, the nucleic acid test is widely adopted to confirm the diagnosis of suspected cases in clinical practice [12]. However, negative results cannot rule out SARS-CoV-2 infection, particularly among those who have epidemiological contact history [13]. Meanwhile, high false-negative results have been reported and the confirmed positive ratio of nucleic acid detection for SARS-CoV-2 was only about 50% [14, 15]. A scoping review reported that the sensitivity of RT-PCR ranged from 57.9% to 94.6% [16]. In particular, nucleic acid tests are subjected to many limitations. First, the detection rate of nucleic acid test varies among different sample types. For example, viral RNA can be present in upper respiratory tract, lower respiratory tract, stool, blood and urine of COVID-19 patients [17], yet not all tissues may be tested positive for SARS-CoV-2 by RT-PCR [15]. Secondly, nucleic acid tests require adequate facilities and instruments, appropriate biosafety measures and skilled laboratory technicians, all of which lead to a significant cost for the test [18, 19]. Thirdly, inappropriate sample collection, storage, transportation, extraction and amplification can all cause false-negative results [18]. Fourthly, the quality and sensitivity of detection kits produced by different companies can greatly affect the detection accuracy [13]. Lastly, different stages of infection in patients [20] along with patients’ history of receiving anti-viral medication (such as anti-HIV drugs) may significantly affect the viral load and even reduce the load to an undetectable level [13]. All these limitations can compromise the accuracy of the nucleic acid test and make it difficult to obtain reliable diagnosis of COVID-19 if using nucleic acid detection alone.

## Antibody detection for SARS-CoV-2

Rapid detection of antibodies is widely used in identification of the causative viral pathogens of respiratory tract viral infections [21]. It was reported that IgM antibody could be detected in patient's serum 3−6 days post-infection and IgG could be detected 8 days post-infection for severe acute respiratory syndrome (SARS) virus [22, 23]. With respect to 2003 SARS virus, it has been shown that specific IgM antibody persists until 2-week post infection, after which its level starts to decrease and eventually disappears [22]. This permits a sufficient window of time for reliable detection of the specific antibodies to SARS-CoV, therefore making this test an effective screening and diagnostic indicator for SARS infection [23]. Given that SARS-CoV-2 belongs to the coronavirus family, the detection of specific antibody for SARS-CoV-2 can serve as an indication of infection [24]. It has been shown that the antibodies against SARS-CoV-2 can be detected in the middle and later stage of the illness [25]. Additionally, several studies [26, 27] have reported that IgM antibodies can appear as early as on the 5th day after symptom onset, peak on the 12th day and then drop slowly, while IgG antibodies are detected around 14 days after symptom onset, reach peak concentrations after day 20, and decrease around the 28th day [25]. A previous study demonstrated that the titre of IgM antibody is usually low, and that IgM antibody only lasts for a short time [28]. As a result, sampling time is critical for accurate assessment of IgM levels [29]. To circumvent this, repetitive testing (5–7 days interval) can be used to monitor the progression of IgM levels. Further, measurement of IgG in combination with IgM can also facilitate the evaluation of disease progression as the production of IgG usually indicates middle or later stage of infection [28]. This is particularly helpful because IgG antibodies show higher titre, last longer and are easier to be detected by immunoassays due to their higher affinity [28]. It is worth noting that because of the long-lasting nature of IgG, pre-existing IgG produced from past infections may complicate the interpretation of the test results of the current diseases. However, it is suggested that an increased IgG titre in convalescent serum of four-fold or higher as compared to that in the acute phase usually reflects a recent infection. Otherwise, a previous infection should be considered [18]. Taken together, high level of IgM antibodies may suggest acute phase of infection, whereas under-detection or low levels of IgM antibodies along with simultaneously rising levels of IgG may suggest middle and later stages of infection.

It has been shown that testing for IgM and IgG antibodies against SARS-CoV-2 provides more clinical value in the diagnosis of suspected COVID-19 patients that have tested negative by other molecular methods [30]. However, these specific antibodies can be detected in patients who have been infected with SARS-CoV-2 regardless of having symptoms or not, patients who have already recovered from COVID-19, and healthy individuals who have received vaccination [31–33]. Therefore, monitoring the dynamics of the antibodies, rather than testing their presence or absence, is more critical for SARS-CoV-2 diagnosis. The levels of specific antibodies vary during different courses of the SARS-CoV-2 infection and can be used to indicate disease progression. As we have mentioned above, it has been shown that high levels of IgM antibodies or an increased IgG titre in convalescent serum that is four-fold or higher relative to that in the acute phase, usually reflects a recent infection [18]. Otherwise, a previous infection should be considered [18]. Consequently, repetitive antibody testing in suspected COVID-19 patients can be used to monitor the seroconversion and/or progression of the antibodies and can provide the most convincing serological evidence to distinguish different disease states. The sensitivity for both IgM and IgG tests ranges between 72.7% and 100%, while the specificity of these tests range between 98.7% and 100%, especially for patients whose disease course lasts ⩾13 days from the disease onset [18, 34]. Further, as compared to the nucleic acid test, the detection of antibody assays are often faster, less expensive, easy-to-use and accessible to staff without laboratory training [9]. Collectively, antibody detection can be used as a powerful testing method in the diagnosis of COVID-19 to complement the viral nucleic acid assays.

The human immune response to SARS-CoV-2 is not well understood. Further, it remains unclear when the levels of specific IgG and IgM antibodies against this virus peak during the course of the disease. This causes several limitations for the utility of antibody for SARS-CoV-2 test. Firstly, false-negative results of the antibody test may result from poor sample quality, low antibody concentrations, individual variations in antibody production and most importantly, inappropriate sampling time. For example, if the antibody test is performed too soon or too late during the course of infection, the antibodies may not have risen to a sufficient level for detection or may have already decreased to an under detection level [9, 35]. Secondly, cross-reactivity of antibody to non-SARS-CoV-2 coronavirus proteins may lead to false-positive result [11]. Therefore, it is critical to combine multiple testing methods to obtain accurate and reliable diagnosis of COVID-19.

Antibody tests for SARS-CoV-2 have been developed rapidly under urgent market demands. The common immunoassay methods used for SARS-CoV-2 antibody detection include enzyme-linked immunosorbent assay (ELISA), chemiluminescence immunoassays (CLIA), fluorescence immunoassays (FIA) and lateral flow immunoassays (LFIA) using immunochromatography [7, 36, 37]. These assays detect IgG and/or IgM antibodies [38, 39] against the receptor binding domain (RBD) of the spike (S) proteins and/or against the nucleocapsid (N) phosphoproteins of the virus in human sera/blood samples and have different sensitivity and specificity. While all display high specificity, ELISA and CLIA-based methods perform better in terms of sensitivity (90–94%) as compared to those using LFIA and FIA which show sensitivities ranging from 80% to 89% [29]. With regard to sensitivity, assays targeting the S antigen appear more sensitive than N antigen-based tests as shown in a meta-analysis of 38 studies [29]. Further, the sensitivities of antibody tests measuring different immunoglobins can be affected by multiple factors, including the timing when the tests are performed. For example, IgG tests exhibit higher sensitivity than IgM tests when the samples were taken longer than 14 days after the onset of symptoms [25, 29]. Therefore, it is recommended to use a combined IgG/IgM test as it performs better in terms of sensitivity than measuring either antibody alone. A simultaneous detection of both IgM and IgG antibodies can be used to identify the stage of the infection and determine the immune status of the individuals [28]. Antibody detection is of great significance for patients who are negative for SARS-CoV-2 nucleic acids, especially for those with an exposure history regardless of whether they present symptoms or not [40]. Moreover, antibody tests are relatively cheap and quick as compared to other tests, thus permitting rapid large-scale screening at points of care (POC) [29]. Further, antibody tests can be used to understand the epidemiology of SARS-CoV-2 infection and to assist in determining the level of humoral immunity in patients [41]. Considering the performance characteristics of every method, a combined IgG/IgM ELISA or CLIA test may be a better and safer choice for individual at this stage of the pandemic [29]. LFIA tests on the other hand are particularly attractive for large seroprevalence studies and can be used as POC tests [29]. Future studies comparing the sensitivity and specificity of different assays using larger sample size will shed light on the optimal ways to administer antibody test for SARS-CoV-2 diagnosis.

## Case reports of bacterial pneumonia misdiagnosed as suspected COVID-19: role of a combination of nucleic acid and specific antibody tests for SARS-CoV-2

As mentioned above, both nucleic acid test and antibody detection methods are critical in the diagnosis of individuals suspected of SARS-CoV-2 infection. However, false-negative results from nucleic acid testing have been increasingly reported whereas antibody test alone cannot confirm or exclude diagnosis. Therefore, a combination of nucleic acid and specific antibody tests for SARS-CoV-2 maybe more effective to diagnose COVID-19 [42], especially for the suspected cases. Here, we present two cases which were initially diagnosed as suspected COVID-19, but finally ruled out by using a combination of nucleic acid and specific antibody tests for SARS-CoV-2.

### Case 1

**Fig. 1.**
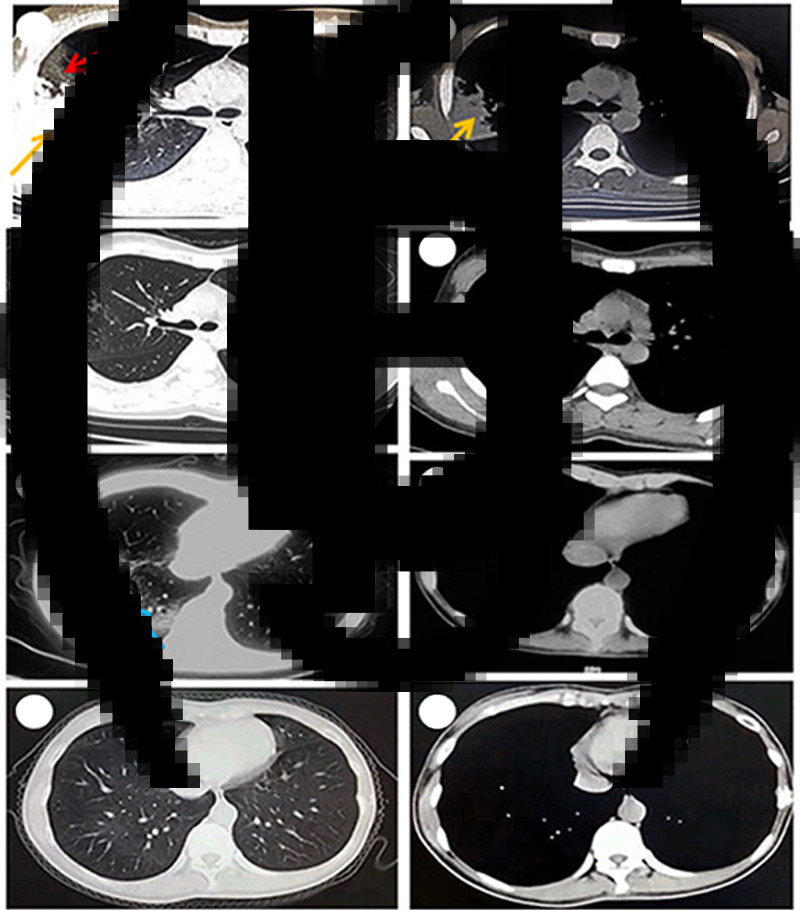
Chest CT of case 1 obtained on 18 February 2020 showing ground glass opacity (a, red arrow) and consolidation (a, b, yellow arrow) in right upper lobe of lung. Chest CT of case 1 obtained on 2 March 2020 showing significant improvement after 1 week' treatment (c, d). Chest CT of case 2 obtained on 10 February 2020 showing ground glass opacity in right lower of lung (e, blue arrow). Chest CT of case 2 obtained on 23 February 2020 showing normal after 10 days of treatment (g, h).

In February 2020, a 23-year-old woman presented to her doctor with a 8-day history of fever, non-productive cough and dyspnoea. She did not have chest pain, chills or weight loss, but experienced dyspnoea after exercise. Her body temperature was as high as above 40 °C. The laboratory test showed that her blood leucocyte count was 5810/mm^3^ with a differential count of 73.6% neutrophils. Her chest computed tomography (CT) scan, performed on 18 February 2020, from another hospital, showed ground glass opacity and consolidation in the right upper lobe of the lung (Fig. 1a, b). She lived in Wuhan city for extended periods of time. The patient was initially diagnosed as suspected COVID-19 and was administered with Moxifloxacin hydrochloride tablets (400 mg/day) and Lianhua Qingwen capsules (a kind of Chinese patent medicine, 12 capsules/day) for six days. However, her symptoms were not improved after the treatment. The patient presented breathlessness on exertion and was then transferred to our hospital, a designated hospital to treat patients with COVID-19. Her family history and previous medical history were unremarkable. At admission, the patient's temperature was 38.6 °C, heart rate was 108 beats per min (bpm), blood pressure, 120/80 mmHg and respiratory frequency, 18 breaths/min. Physical examination of the chest revealed exaggerated breath-sounds on the right upper chest, but without wheezes or moist rales. Blood routine examination and procalcitonin (PCT) exhibited normal. Two haemocultures and two sputum cultures were negative for microorganisms. Serologic tests for respiratory syncytial virus, adenovirus, influenza A virus, influenza B virus, parainfluenza virus, Epstein−Barr virus, cytomegalovirus, *Mycoplasma pneumoniae*, *Chlamydia pneumoniae*, *Legionella pneumophila* were negative. Nasopharyngeal swab specimens were collected twice from the patient (24-h interval) to perform nucleic acid test for SARS-CoV-2 and both were negative. Meanwhile, serum-specific IgM and IgG antibodies to SARS-CoV-2 were screened for twice by a SARS-CoV-2 IgM and IgG chemiluminescence immunoassay (CLIA) kit against S protein and N protein antigen, respectively (Yahuilong Biotechnology, Shenzhen, China, lot number: 20200101) and the results were all negative. The patient was quarantined in single room and was treated with intravenous cefoperazone-sulbactam (6 g/day) and moxifloxacin (400 mg/day). Her symptoms and her chest CT scan showed significant improvement after 1 week of treatment (Fig. 1c, d). The patient was thus diagnosed as bacterial pneumonia and had recovered from the treatment.

### Case 2

On 10 February 2020, a 30-year-old woman complained of fever and productive cough for 3 days with unknown causes. She reported that she sometimes spat white sputum and did not have chest pain, chills or dyspnoea. Her highest body temperature was over 39 °C. Her chest CT scan showed ground glass opacity in right lower lobe of lung (Fig. 1e, f) but her blood routine examination was normal. She was treated with cefaclor capsules (1.5 g/day) for four days without relief of her symptoms before being admitted to our hospital as suspected case of COVID-19. She lived in Wuhan for a long time. Her previous medical history and family history was unremarkable. At admission, the patient's temperature was 39.4 °C, heart rate was 85 bpm, blood pressure, 115/65 mmHg and respiratory frequency, 16 breaths/min. Her lung breath sounded clear without wheezes or moist rales. The results of PCT and arterial blood gas analysis were normal. Her blood routine test at admission showed that blood leucocyte count was 5340/mm^3^ with a differential count of 76.8% neutrophils. Two haemocultures and two sputum cultures were negative, but sputum smears showed Gram-positive bacteria and leucocyte count >20/low-power microscope field. Serological test for influenza virus A and B, parainfluenza of type 1, 2 and 3, respiratory syncytial virus, adenovirus, *Mycoplasma pneumonia, Chlamydophila pneumonia, Legionella pneumophila* and Q Rickettsia was performed and the results were all negative. The SARS-CoV-2 nucleic acid was tested twice from nasopharyngeal swab samples by real-time RT-PCR (24-h interval) and both were negative. Additionally, regarding her serum antibody tests for SARS-CoV-2, two independent tests for specific IgM and IgG antibodies were performed on a SARS-CoV-2 IgM and IgG CLIA kit (Yahuilong Biotechnology, Shenzhen, China, lot number: 20200101) and the levels were all negative. Specifically, her specific IgM antibody levels were 0.58 and 0.42 AU/ml (normal value <10 AU/ml), respectively, and her specific IgG antibody levels were 0.94 and 0.37 AU/ml (normal value <10 AU/ml), respectively. The patient was treated in our hospital with Moxifloxacin hydrochloride tablets (400 mg/day) and amoxicillin capsules (1.5 g/day) in isolation ward. Her symptoms were improved markedly in 5 days. The chest CT scan showed normal after 10 days of treatment (Fig. 1g, h). She had fully recovered and was discharged as bacterial pneumonia.

Both these patients presented fever and respiratory symptoms when admitted to our hospital. Their complete blood white-cell count was normal but their chest CT scan showed pulmonary infiltration. They were tested for common respiratory pathogens but the results were all negative. Both patients have been living in Wuhan for a long time. Given that their symptoms, chest imaging and lab tests results and residential history match the diagnostic criteria of suspected COVID-19 cases, these patients were initially diagnosed as suspected COVID-19 cases and were treated in isolated single room in our hospital to avoid cross infection. According to WHO interim guidance, the confirmed cases must have positive results of nucleic acid test for SARS-CoV-2 [7]. However, both patients were tested negative for SARS-CoV-2 through nucleic acid test twice. Meanwhile, serum-specific antibodies for SARS-CoV-2 were also performed twice and were all negative. Furthermore, they both recovered after antibiotic treatment. Therefore, our patients were finally diagnosed as bacterial pneumonia.

## Conclusion

In conclusion, COVID-19 often exhibits non-specific clinical features and chest imaging appearances, which are difficult to be distinguished from many other infectious pulmonary disorders. As a result, bacterial pneumonia that causes pulmonary infiltration can be easily misdiagnosed as COVID-19 during the epidemic. Therefore, a combination of nucleic acid and specific antibodies tests will be helpful and critical for identifying confirmed COVID-19 patients and excluding suspected cases.

## Data Availability

The datasets analysed during the current study are available from the corresponding author on reasonable request.
